# Morphological variations in a widely distributed Eastern Asian passerine cannot be consistently explained by ecogeographic rules

**DOI:** 10.1002/ece3.8208

**Published:** 2021-10-12

**Authors:** Chun‐Cheng Lee, Yuchen Fu, Chia‐fen Yeh, Carol K. L. Yeung, Hsin‐yi Hung, Chiou‐Ju Yao, Pei‐Jen Lee Shaner, Shou‐Hsien Li

**Affiliations:** ^1^ School of Life Science National Taiwan Normal University Taipei Taiwan; ^2^ Novogene Bioinformatics Institute Beijing China; ^3^ Department of Biology National Museum of Natural Science Taichung Taiwan

**Keywords:** Allen's rule, Bergmann's rule, ecogeographic rule, Gloger's rule, morphometrics, plumage coloration

## Abstract

Ecogeographic rules that describe quantitative relationships between morphologies and climate might help us predict how morphometrics of animals was shaped by local temperature or humidity. Although the ecogeographic rules had been widely tested in animals of Europe and North America, they had not been fully validated for species in regions that are less studied. Here, we investigate the morphometric variation of a widely distributed East Asian passerine, the vinous‐throated parrotbill (*Sinosuthora webbiana*), to test whether its morphological variation conforms to the prediction of Bergmann's rule, Allen's rules, and Gloger's rule. We at first described the climatic niche of *S. webbiana* from occurrence records (*n* = 7838) and specimen records (*n* = 290). The results of analysis of covariance (ANCOVA) suggested that the plumage coloration of these parrotbills was darker in wetter/warmer environments following Gloger's rule. However, their appendage size (culmen length, beak volume, tarsi length) was larger in colder environments, the opposite of the predictions of Allen's rule. Similarly, their body size (wing length) was larger in warmer environments, the opposite of the predictions of Bergmann's rule. Such disconformity to both Bergmann's rule and Allen's rule suggests that the evolution of morphological variations is likely governed by multiple selection forces rather than dominated by thermoregulation. Our results suggest that these ecogeographic rules should be validated prior to forecasting biological responses to climate change especially for species in less‐studied regions.

## Graphical abstract

The morphometric variations of the vinous‐throated parrotbill (*Sinosuthora webbiana*) are not conform to predictions of Bergmann’s and Allen’s rules. This figure shows the positive relationships between temperature variables (bio1 and bio5) and wing length which against the prediction of Bergmann’s rule. Also, the negative relationships between temperature variables (bio1 and bio6) and beak size do not match the prediction of Allen’s rule.

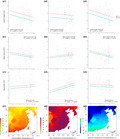

## 1 INTRODUCTION

Classic ecogeographic rules (Millien et al., 2006; Scheffers et al., 2016; Yom‐Tov & Geffen, 2011) summarize the associations between variations in climate and morphological traits. For instance, endotherms living in colder regions tend to be larger (Bergmann's rule, Bergmann, 1847) and have shorter appendages (e.g., limbs or beaks; Allen's rule, Allen, 1877) than those living in warm regions to reduce the heat loss; endotherms in humid and/or warm climates tend to have more heavily pigmented feathers, hair, or skins than those in dry and/or cold environments (Gloger's rule, Gloger, 1833; Mayr, 1942; Rensch, 1938). These ecogeographic rules describe how morphological traits of a species are constrained by climatic environments and its physiological requirements. In addition, in the last two centuries, anthropogenic disturbances have significantly altered the global climate by raising temperatures (Crowley, 2000; Solomon et al., 2009), redistributing precipitation patterns (Trenberth, 2011; Zhang et al., 2007), and increasing the frequency of extreme climatic events (Emanuel, 1987). Such changes substantially impact the physiology, distribution, and phenology of organisms, the biological interactions within local communities, local adaptation (Hoffmann & Sgrò, 2011; Hughes, 2000; Parmesan, 2006; Scheffers et al., 2016; Walther, 2010; Walther et al., 2002), and even species extinction (Cahill et al., 2013; Garcia et al., 2014; Thomas et al., 2004). As anthropogenic climate change is expected to be accelerated in the near future, the ecogeographic rule might provide an adaptive aspect to foresee how organisms would respond morphologically to ongoing climate change (Tian & Benton, 2020) or extreme climate events (Danner & Greenberg, 2015) in ways analogous to their adaptation to environmental gradients.

A large body of empirical data supports various ecogeographic rules (e.g., Meiri, 2011; Salewski & Watt, 2017 for Bergmann's rule; Betti et al., 2015; Laiolo & Rolando, 2001; VanderWerf, 2012; Yom‐Tov & Yom‐Tov, 2005 for Allen's rule; and Delhey, 2019 for Gloger's rule) although it is not uncommon to document exceptions to these rules (e.g., Núñez‐Zapata et al., 2018; Riemer et al., 2018; Tattersall et al., 2017). Moreover, most of these studies focused on European and North American species (Betti et al., 2015; Delhey, 2019; Laiolo & Rolando, 2001; Meiri, 2011; Salewski & Watt, 2017; VanderWerf, 2012; Yom‐Tov & Yom‐Tov, 2005), and the rules remain to be tested against evolutionary lineages endemic to other geographic regions. Validating these ecogeographic rules in taxa from regions that have been less studied is critical for assessing their universality.

Here, we present the range‐wide patterns in morphometric traits and plumage coloration of a widely distributed East Asian endemic passerine, the vinous‐throated parrotbill (*Sinosuthora webbiana*), and their associations with climate, explicitly testing Bergmann's rule, Allen's rule, and Gloger's rule. The vinous‐throated parrotbill is widely distributed in open‐wooded habitats ranging from northern Indochina to southern Siberia and from the eastern edge of the Tibetan Plateau to coastal China and the island of Taiwan (Robson, 2020; Table 1). One island endemic, *S. w. bulomacha*, and five mainland subspecies (Robson, 2020) have been described. Although *S. webbiana* is common and probably one of the most widely distributed avian species in East Asia, its morphological variations have been poorly described: Only limited numbers of skin specimens have been measured and documented (Yen & Severinghaus, 2017; Zheng, 1987) with no quantification of coloration. Limited information suggests morphological variations within its range (Robson, 2020; Zheng, 1987), where the northernmost subspecies (*S. w. mantschurica*) found in southern Siberia and Northeast China tend to have shorter culmen and paler plumage coloration (Zheng, 1987). Given that *S. webbiana* is nonmigratory, it serves as an ideal system for investigating the associations between morphologies and the local climatic environment.

**TABLE 1:** Results of ANCOVAs examining relationships between morphometric traits and climatic variables

Response	Fixed effect	Confidence interval of slope		
		Lower limit	Median	Upper limit
Culmen length	**bio1**	**−0.0399**	**−0.0239**	**−0.0078**
	bio5	−0.0259	−0.0107	0.0044
	**bio6**	**−0.0253**	**−0.0162**	**−0.0072**
Beak volume	**bio1**	**−0.1163**	**−0.0681**	**−0.0200**
	**bio5**	**0.0095**	**0.0578**	**0.1057**
	**bio6**	**−0.0912**	**−0.0631**	**−0.0350**
Tarsus length	bio1	−0.0172	−0.0004	0.0154
	bio5	−0.0136	0.0058	0.0245
	bio6	−0.0120	−0.0026	0.0068
Wing length	**bio1**	**0.0001**	**0.0764**	**0.1550**
	**bio5**	**0.1910**	**0.2775**	**0.3636**
	bio6	−0.0533	−0.0095	0.0357

Note

Sex was treated as a covariate. Lower limit (2.5%) and upper limit (97.5%) show the 95% confidence interval (CI) of slope from 10,000 replicates. Statistical significance was determined by whether the 95% CI of the estimated slope from resampled datasets includes zero. Significant results are in bold. bio1: annual mean temperature (°C), bio5: maximum temperature of warmest month (°C), bio6: minimum temperature of coldest month (°C).

In this study, we first describe the climatic niche of *S. webbiana* based on climatic data from sites where the species occurs and proceed by testing three well‐known ecogeographical rules, namely Bergmann's rule, Allen's rule, and Gloger's rule. We predicted that (1) appendage size (culmen length, beak volume, tarsus length) of *S. webbiana* would be positively associated with temperature (Allen's rule); (2) body size (wing length as a proxy, see Sullivan et al., 2019) of *S. webbiana* would be negatively correlated with temperature (Bergmann's rule); (3) melanin‐based pigmentation of plumage would be positively associated with precipitation and/or temperature (Gloger's rule). Our results show that the variation of size and coloration of this widely distributed East Asian passerine cannot be consistently predicted by these ecogeographic rules. It highlights that evolution of morphological traits could be influenced by selection forces other than the demand of thermal regulation and suggests that these rules should be validated prior to forecasting biological responses to climate change.

## 2 MATERIALS AND METHODS

### 2.1 Geographic coverage

The geographic coordinates of a total of 18,306 occurrence records of *S. webbiana* on the Asian mainland were obtained from literature, the georeferences of skin specimens in museum archives, a public database (Global Biodiversity Information Facility, GBIF, http://www.GBIF.org), and observation records contributed by community scientists (China Bird Report, http://birdreport.cn; eBird, http://eBird.org). The island subspecies were excluded from the current study because the body size of vertebrates on islands tends to be larger or smaller than their continental relatives (island rule, Lomolino, 2005; Valen, 1973). The geographic coverage of our data is approximate to the species’ distribution range depicted by Robson (2020).

### 2.2 Range of climatic conditions occupied by *S*. *webbiana*

To depict the range of climatic conditions in which *S. webbiana* occurred, we used the six bioclimatic variables most associated with the three geographic rules: annual mean temperature (bio1), maximum temperature of warmest month (bio5), minimum temperature of coldest month (bio 6), annual precipitation (bio12), precipitation of wettest quarter (bio16), and precipitation of driest quarter (bio 17) (Danner & Greenberg, 2015; Millien et al., 2006). We used the geographic coordinates of the occurrence records to extract the six selected bioclimatic variables from the WorldClim dataset (http://www.worldclim.org/, Fick & Hijmans, 2017), which was based on the average of the years 1970–2000 at a 30‐arc‐second (~1 km^2^) spatial resolution. The geospatial extraction was performed with raster package v3.4‐5 in R (Hijmans, 2020).

### 2.3 Morphometric measurements

In order to test hypotheses associated with Allen's rule and Bergmann's rule, we measured skin specimens of 155 males and 135 females archived in the Institute of Zoology, Chinese Academy of Sciences, China, the Kunming Institute of Zoology, Chinese Academy of Sciences, China, the Sichuan Agriculture University, China, and National Museum of Natural History, USA. We used a digital caliper (model number CD‐8’CS; MitutoyoCorp.) with a precision of ±0.02 mm to measure five morphometric traits: beak depth (distance between commissure and point of beak), beak width (distance between the commissures on both sides), and lengths of culmen, tarsus, and wing. We inferred the sixth morphometric trait, beak volume, using the cone volume formula (1/3× ((beak width)/2)^2 ^× beak gap length). Because weights of specimens are generally lacking, and the mechanics analysis showed positive correlation between wing humerus length and body weight (Sullivan et al., 2019), we used wing length as the surrogate of body size. All measurements were taken from the right side of birds if possible, in units of mm. All morphometric measurements were obtained by Chia‐Fen Yeh.

### 2.4 Coloration measurements

To test the hypothesis associated with Gloger's rule, plumage coloration was quantified from 43 males and 58 females in good plumage condition. The coloration was measured by the USB2000 spectrometer (Ocean Optics) with a HL2000 deuterium‐halogen light source (Ocean Optics) and a R600‐7‐UV/125F probe (Ocean Optics). A white standard (Labsphere) was used as the white reference. Three body parts, crown, cheek, and wing, were measured. These parts are mainly in brown and represent coloration of dorsal and lateral sides of body. The stripes on breast plumage could interrupt the measurement, and the back is mostly covered by wings; thus, we measured these three parts to represent coloration of *S. webbiana*. Each part was measured three times, and the mean spectra were then calculated. Spectral data were summarized as measures of total brightness, chroma, and hue all of which are positively correlated with levels of total melanin expression in feathers (McGraw et al., 2005). Total brightness was defined as the average reflectance observed within a range of 300–700 nm. Chroma was the proportion of the total brightness which fell in the range of 550–700 nm (the range for brown color). Additionally, as the reflectance of melanin steadily increases from 300 to 700 nm (Figure S1) and shows no spectral peaks, and there is no significant UV reflectance between 300 and 400 nm, we calculated the hue as the slope of reflectance regressed against wavelength in the 400–700 nm range for melanin‐based color expression (Galván & Wakamatsu, 2016). Finally, we averaged the brightness, chroma, and hue of these three body parts respectively to represent the body color for each individual (Senar et al., 2003). Coloration measurements were obtained by Hsin‐yi Hung and Chiou‐Ju Yao.

### 2.5 Statistics

We analyzed morphometric and coloration datasets separately. To control the effect of body size on the morphometric traits, we regressed culmen length, tarsus length, and beak volume against wing length, a proxy for body size (Sullivan et al., 2019), and extracted the residuals to serve as body size‐independent traits in following analysis. We performed analysis of covariance (ANCOVA) to control for potential sexual dimorphism and explore sex‐specific climate‐trait associations. Because the interactions between bioclimatic variables and sex are not significant in all analyses (all *p *> .05), we ran ANCOVA for each trait—bioclimatic variable combination (each trait as the response variable, and each bioclimatic variable as the fixed effect) with sex as the covariate. We included only one bioclimatic variable at a time because these variables tend to have high correlations (Table S2).

To reduce pseudoreplication in morphometric and coloration data, we used locality as the sampling unit (1–19 individuals sampled from each of the 114 localities for morphometric data and 41 localities for coloration data; Table S1). For localities with multiple individuals, we randomly selected one individual from each to ensure even sample size across all localities. We then replicated ANCOVA 10,000 times, each with the randomly selected samples, to obtain 95% confidence interval (CI) of the estimated slope. The 95% CI of the estimated slope from resampled datasets includes zero was considered to have no significant relationship between morphometric traits and bioclimatic variables. All statistical analyses were performed with R version 4.0.1 (R Core Team, 2020).

## 3 RESULTS

### 3.1 Range of climatic conditions occupied by *S*. *webbiana*

**FIGURE 1:**
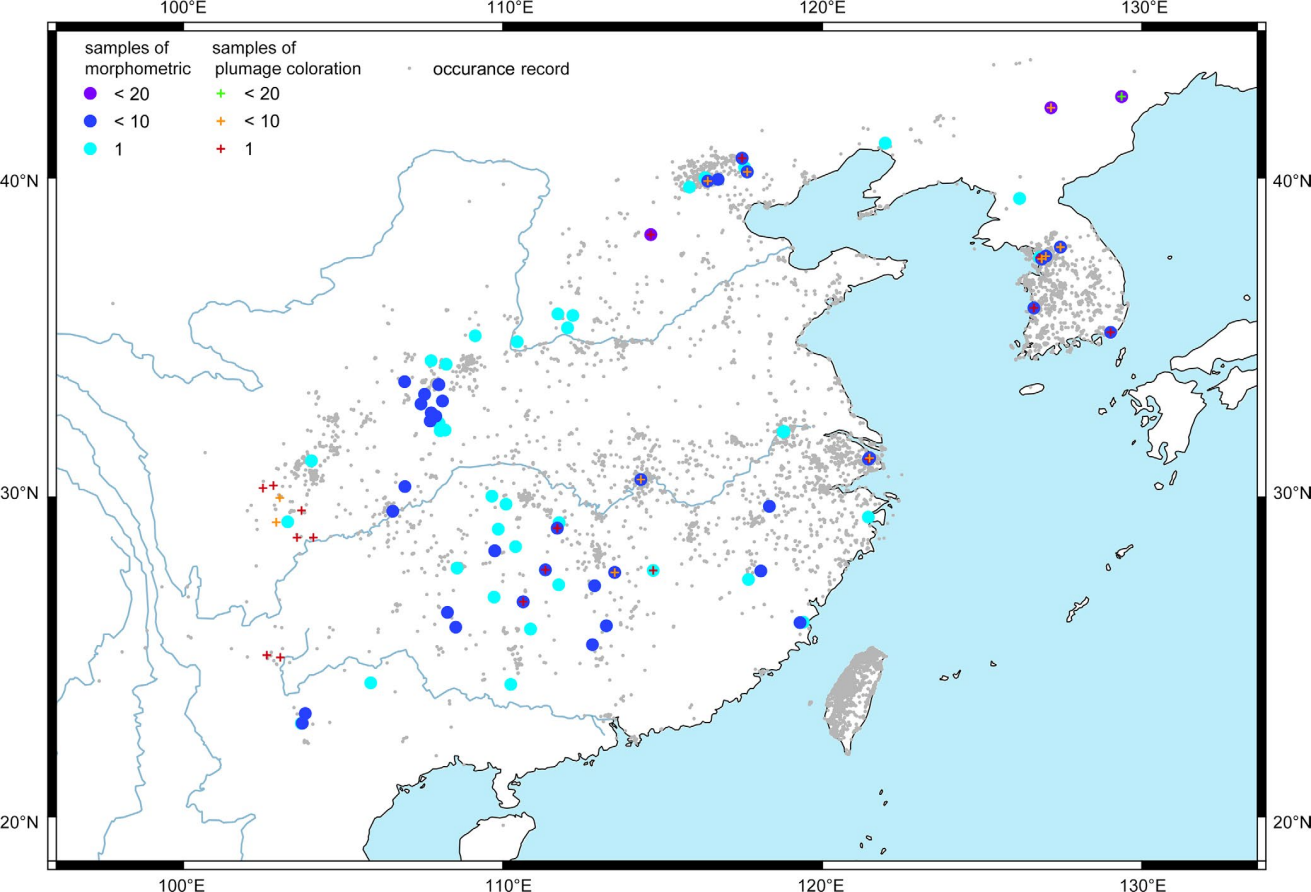
Localities of the vinous‐throated parrotbill specimens used in the current study. The localities for morphometric, plumage coloration, and occurrence records are shown in solid circles, crosses, and grey dots, respectively

**FIGURE 2:**
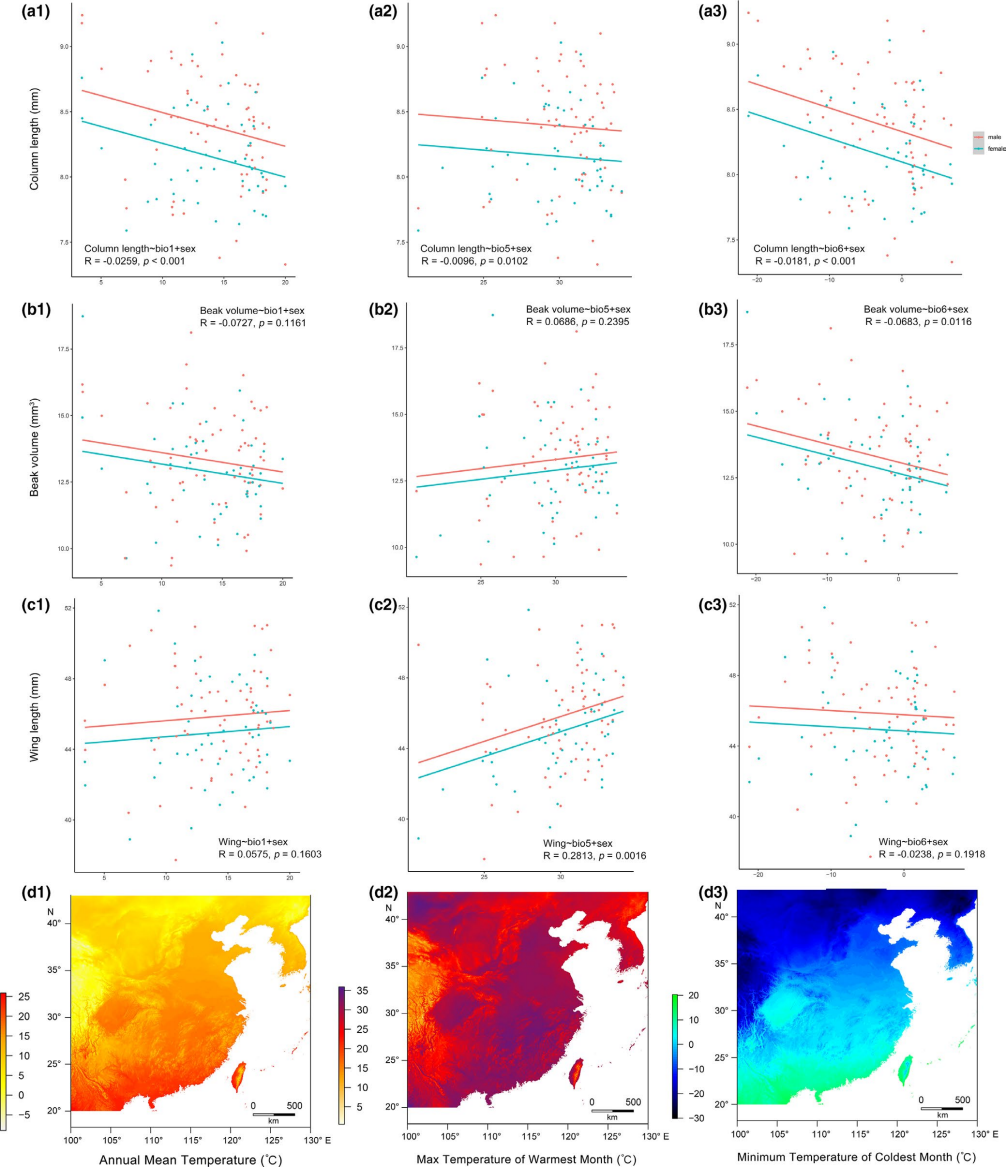
Correlations between morphometric traits (*n* = 114) and bioclimatic variables: (a) culmen length; (b) beak volume; (c) wing length; and (d) heat maps of each bioclimatic variable in East Asia: annual mean temperature (*bio1*), max temperature of warmest month (bio5), minimum temperature of coldest month (bio6). The solid lines are the regression lines (red: male, blue: female) showing the relationships between traits and bioclimatic variables. This figure shows the result of one random resampled replica

Occurrence records were collected from 7,838 unique localities (WorldClim grids) that span about 24 degrees in latitude (21.6–45.3°N, Figure 1). *S. webbiana* occupies a broad range of temperature and precipitation niches (Figure 2). For example, the annual mean temperature (bio1) ranged from −0.8 to 24.3°C, and the annual precipitation (bio12) was in the range of 307.8 to 2381.3 mm. At the extremes, the maximum temperature of warmest month (bio5) could be up to 34.9°C and the minimum temperature of coldest month (bio6) could be as low as −27.1°C. The complete climatic niche (ranges of all six bioclimatic variables) according to the occurrence records is shown in Table S3.

For the 290 skin specimens we measured, the morphometric dataset contains 114 independent localities, and the plumage coloration dataset is composed of 41 of them. For localities where skin specimens were collected, the annual temperature (bio1) ranged from 2.9 to 20.6°C (2.9–17.7°C for the plumage coloration dataset), and the annual precipitation (bio12) was in the range of 471.9–1837.mm (471.9–1634.7 mm for the plumage coloration dataset). The lowest and highest maximum temperature (bio5) were 20.9°C and 33.8°C, respectively (15.7°C and 33.2°C for the plumage dataset); the lowest and highest minimum temperature (bio6) were −23.6°C and 8.2°C, respectively (−23.6°C to 4.4°C for the plumage coloration dataset). Ranges of all six bioclimatic variables according to specimens’ records are listed in Table S4.

### 3.2 Morphometric analysis

The descriptive statistics of the morphometric measurements are listed in Table S5. *Sinosuthora webbiana* showed a clear sexual dimorphism in culmen, tarsus, and wing length (Figure S2). We found that the culmen length and the beak volume were negatively correlated with annual mean temperature (bio1) and minimum temperature of coldest month (bio6) (Table 1), and the beak volume was positively correlated with maximum temperature of warmest month (bio5) (Table 1; Figure 2). The length of tarsus was unrelated to the three bioclimatic variables (Table 1). The wing length was positively correlated with annual mean temperature (bio1) and maximum temperature of warmest month (bio5) (Table 1; Figure 2). Traits after control of the body size still showed the similar results (Table S6). Therefore, body size did not appear to affect the relationship between morphometric traits and climatic variables we observed here.

### 3.3 Plumage coloration analysis

**FIGURE 3:**
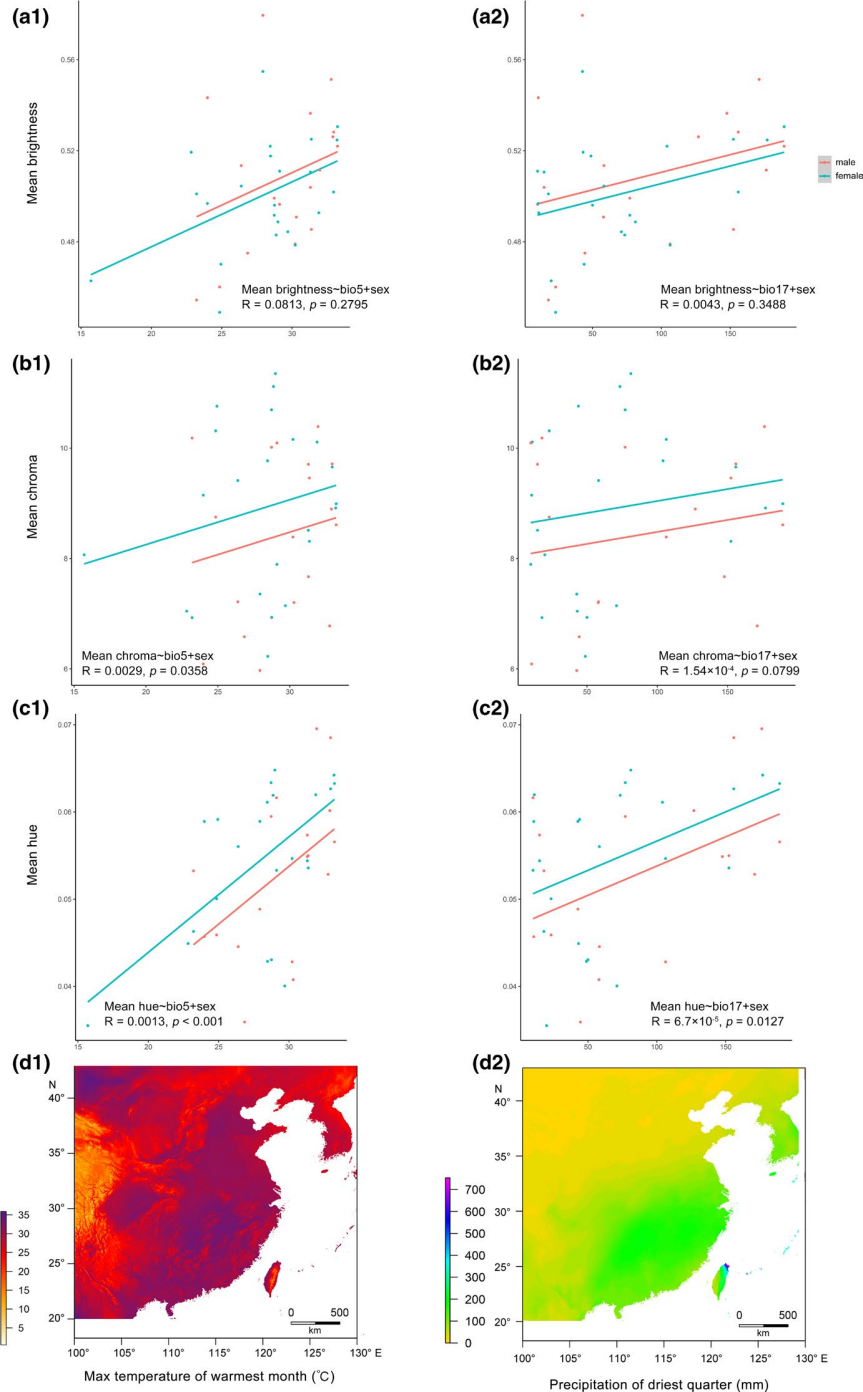
Correlations between plumage coloration (*n* = 41) and bioclimatic variables: (a) Brightness; (b) chroma; (c) hue; and (d) heat maps of each bioclimatic variable in East Asia: max temperature of warmest month (bio5) and precipitation of driest quarter (bio17). The solid lines are the regression lines (red: male, blue: female) showing the relationships between traits and bioclimatic variables. This figure shows the result of one random resampled replica

The descriptive statistics of the plumage coloration measurements are listed in Table S5. *Sinosuthora webbiana* showed a clear sexual dimorphism in brightness and chroma (Figure S2). Brightness was negatively correlated with annual mean temperature (bio1) and minimum temperature of coldest month (bio6) (Table 2) and positively correlated with maximum temperature of warmest month (bio5) (Table 2; Figure 3). Chroma was positively correlated with three temperature variables (annual mean temperature, bio1, maximum temperature of warmest month bio5, and minimum temperature of coldest month, bio6), and two precipitation variables (annual precipitation, bio12, and precipitation of driest quarter, bio17) (Table 2; Figure 3). Hue was positively correlated with annual mean temperature (bio1), maximum temperature of warmest month (bio5), precipitation of wettest quarter (bio16), and precipitation of driest quarter (bio17) (Table 2; Figure 3).

**TABLE 2:** Results of ANCOVAs examining relationships between plumage coloration and climatic variables

Response	Fixed effect	Confidence interval of slope		
		Lower limit	Median	Upper limit
Brightness	**bio1**	**−0.0959**	**−0.0512**	**−0.0066**
	**bio5**	**0.0016**	**0.0453**	**0.0889**
	**bio6**	**−0.0746**	**−0.0486**	**−0.0227**
	bio12	−5.5 × 10^−4^	−1.2 × 10^−4^	3.1 × 10^−4^
	bio16	−1.5 × 10^−3^	−3.4 × 10^−4^	8.0 × 10^−4^
	bio17	−2.1 × 10^−4^	2.3 × 10^−3^	4.8 × 10^−3^
Chroma	**bio1**	**0.0014**	**0.0023**	**0.0032**
	**bio5**	**0.0021**	**0.0029**	**0.0036**
	**bio6**	**6.6 × 10^−4^**	**1.2 × 10^−3^**	**1.7 × 10^−4^**
	**bio12**	**7.5 × 10^−6^**	**1.5 × 10^−5^**	**2.3 × 10^−5^**
	bio16	−2.0 × 10^−5^	9.9 × 10^−7^	2.2 × 10^−5^
	**bio17**	**1.1 × 10^−4^**	**1.6 × 10^−4^**	**2.3 × 10^−4^**
Hue	**bio1**	**1.3** × 10^−4^	**3.8** × 10^−4^	**6.2** × 10^−4^
	**bio5**	**9.8 × 10^−4^**	**1.2 × 10^−3^**	**1.4 × 10^−3^**
	bio6	−9.5 × 10^−5^	5.2 × 10^−5^	1.9 × 10^−4^
	**bio12**	**5.3 × 10^−7^**	**2.7 × 10^−6^**	**5.0 × 10^−6^**
	bio16	−1.1 × 10^−5^	−5.3 × 10^−6^	2.8 × 10^−7^
	**bio17**	**4.8 × 10^−5^**	**6.3 × 10^−5^**	**7.8 × 10^−5^**

Note

Sex was treated as a covariate. Lower limit (2.5%) and upper limit (97.5%) show the 95% confidence interval (CI) of slope from 10,000 replicates. Statistical significance was determined by whether the 95% CI of the estimated slope from resampled datasets includes zero. Significant results are in bold. bio1: annual mean temperature (°C), bio5: maximum temperature of warmest month (°C), bio6: minimum temperature of coldest month (°C), bio12: annual precipitation (mm), bio16: precipitation of wettest quarter (mm), bio17: precipitation of driest quarter (mm).

## 4 DISCUSSION

To evaluate Gloger's rule, ideally coloration of the entire body should be used. However, this may not be practical for some species, such as *S. webbiana*, given that their breast plumage is stripped and their back is mostly covered by wings. Our results demonstrate that the geographic variation of plumage coloration of *S. webbiana* largely supports Gloger's rule. Furthermore, we found that, except that the beak volume was positively correlated with maximum temperature of warmest month, most of morphometric traits related to thermoregulation such as beak volume, tarsus length, and body size cannot be fully explained by Allen's or Bergmann's rule. Though the mechanics analysis showed positive correlation between wing humerus length and body weight (Sullivan et al., 2019), wing length could also be used as appendages to evaluate Allen's rule (Gutiérrez‐Pinto et al., 2014). Since weights of specimens are generally lacking, and the head size was positively correlated with body mass in *S. webbiana* (Rao et al., 2018), we recommend using head size (skull) in future studies to explore the size–climate relationship while examining Bergmann's rule in *S. webbiana*.

### 4.1 Conformity to Gloger's rule in *S*. *webbiana*

Gloger's rule proposes that the deposition of total melanin increases with humidity and temperature (Rensch, 1938). However, 43 quantitative studies up to 2019 (Delhey, 2019) suggest that Gloger's rule holds for humidity but not temperature (Delhey, 2019). Our results, on the other hand, provided support for both humidity and temperature: The crown plumage coloration of *S. webbiana* was darker at wetter localities, and the plumage coloration of the crown and cheek was darker at warmer localities (Figure 3; Table 2). We caution that due to the positive correlations between temperature and precipitation in the current study (Table S1) as well as across East Asia, intraspecific diversification of plumage coloration in parrotbills could be influenced by either temperature, precipitation, or both.

There are several alternative explanations for the association between darker plumage and increased temperature and precipitation. First, darker plumage as an adaptation to predation pressure by enhancing camouflage in dense vegetation (Zink & Remsen, 1986), which is positively associated with precipitation in China (Zhang et al., 2003), or enhancing background‐matching in snow, as recently reported that the northern East Asia (approximately north of 33°N) temperature tends to be low with snow in winter (Li et al., 2015). Therefore, the paler plumage in the northern range of the parrotbill could provide better background‐matching in winter. Second, darker plumage as an antiparasitic strategy by strengthening feathers with increased melanin production (Bonser, 1994) to reduce degradation by bacteria which have higher keratinolytic abilities in humid environments (Burtt & Ichida, 2004). Third, darker plumage as a by‐product of selection operating on other traits and having pleiotropic effects. More specifically, the genes coding for the melanocortin receptor (MC1R) and its ligands, which affect the deposition of melanin pigments, have been found to have a pleiotropic effect on traits such as sexual activity, aggressiveness, sensitivity to stress, energy balance and anti‐inflammatory, antipyretic, and anti‐oxidative responses (Ducrest et al., 2008). Therefore, darker plumage coloration in hot/humid environments could also be a side effect of selection operating on these traits along the temperature/humidity gradient. For instance, it has been shown that parasites can develop more quickly in warm environments (Franke et al., 2017) and that wildlife immunocompetence is higher in hot environments (Jackson et al., 2020). Therefore, the darker plumage coloration in *S. webbiana* might be attributed to selection for high immunocompetence in high temperatures for better parasite resistance, rather than to selection forces directly operating on plumage color.

However, we also found positive association between brightness and maximum temperature of warmest month (bio5), which was contrary to the correlations with other temperature variables (annual mean temperature and minimum temperature) and the prediction of Gloger's rule. Thermoregulatory advantage of darker coloration in colder places had been proposed mainly for ectotherm (Bogert, 1949; Clusella Trullas et al., 2007), and some research also discovered this pattern in bird and mammals (Hamilton & Heppner, 1967; Wacker et al., 2016) or lighter plumage in warmer environment (Ribot et al., 2019). Our data showed *S. webbiana* had higher plumage brightness in environment with warmer summer but not lower plumage brightness in environment with colder winter. This may indicate that thermoregulation plays a more important role on plumage brightness in hotter environment for avoiding heat absorption than colder environment for reducing energy expenditure. To fully understand the mechanistic underpinning of any observed association between plumage coloration and climate, we suggest future studies to focus on disentangling the roles of climate, natural enemies, and pleiotropy in shaping variations of plumage coloration.

### 4.2 Disconformity to Allen's rule and Bergman's rule

All else being equal, the surface area‐to‐volume ratio of body appendages determines the rate of heat dissipation for endothermic animals. Allen's rule (Allen, 1877) predicts that endotherms living in cold climates should have appendages with small surface area‐to‐volume ratios (e.g., smaller beaks, shorter tarsi) that minimize heat loss and thermoregulation cost. At both intraspecific (Fan et al., 2019; Nudds & Oswald, 2007; Tattersall et al., 2017) and interspecific (Friedman et al., 2017; Laiolo & Rolando, 2001; Symonds & Tattersall, 2010; VanderWerf, 2012) levels, it is commonly found that birds living in cold environments have smaller beaks and shorter tarsi. However, in the case of *S. webbiana*, only maximum temperature of warmest temperature (bio5) was positively correlated with beak volume, which conforms to Allen's prediction. It supports that beak of the parrotbill could serve as the heat radiator in summer. Culmen length and beak volume are negatively associated with annual mean temperature (bio1) and minimum temperature of coldest month (bio6) and tarsus length is not correlated with temperature, suggesting that thermoregulation is not a major selection force determining appendage morphology.

The avian beak is multifunctional, serving not only as a heat radiator (Tattersall et al., 2017), but also as a tool for capturing and processing food (Boag & Grant, 1981; Cooney et al., 2017), building nests (Collias & Collias, 1964), preening and parasite control (Clayton et al., 2005), and sound production (Podos, 2001; Podos & Nowicki, 2004). As such, the diversification of beak morphology could be driven by factors such as the food diversity and availability, parasitism, vocal communication including vocalization‐based sexual selection. For instance, the larger beaks of seed‐cracking species provide greater bite force (Herrel et al., 2005) to allow for more efficient consumption of larger and harder seeds (Gibbs & Grant, 1987; Smith & Girman, 2000), and in Darwin's finch, individuals with larger beaks tend to produce lower frequency sounds (Podos, 2001; Podos & Nowicki, 2004). For *S. webbiana*, the beak size and the maximum bite force had been found to be varied among populations: The northern population had stronger bite force than the southern one (Rao et al., 2018). Moreover, the maximum bite force was positively associated with bill depth (W. Liang, personal communication). It had been proposed that individuals with larger beaks and larger bite force should be able to exploit larger and more variable food items (Gomes et al., 2018; Lefebvre et al., 1997). Although *S. webbiana* mainly feeds on plant seeds (Robson, 2020; Severinghaus, 1991), when seeds become scarce in Taiwan's winter, they have been seen tearing open grass stems to feed on the worms inside (Yen & Severinghaus, 2017). Therefore, it is possible that evolution of the finch‐like sturdy beak, as in case of *S. webbiana*, is shaped by the variety and richness of food items available in the varied climatic environments other than thermoregulation.

Similar to Allen's rule, Bergmann's rule proposes that the rate of heat dissipation is a major driving force for the evolution of body size: Endotherms with larger bodies have smaller surface area‐to‐volume ratios and should be favored in cold environments (Bergmann, 1847). Although Bergmann's rule has been supported in various studies (Meiri, 2011; Salewski & Watt, 2017), body size is not simply determined by the rate of heat dissipation alone. For instance, larger birds may have advantage in place with higher food availability and diversity of size (Wilson, 1975). Also, speed of locomotion could serve as evolutionary constraints on the body size of endotherms, as it has been found that larger mammals and birds have greater oxygen consumption per mass at the same speed of locomotion (Taylor et al., 1982). Since *S. webbiana* mainly shuttle among shrubs, vegetation density could act a role to influence their body size. Though the smaller bird may get more advantage in denser vegetation for moving, perching, nesting, and protection (Clark, 1979), a global research of avian body size showed that median body mass of an avian assemblage is positively associated with vegetation density (Olson et al., 2009). Because the southern East Asia have denser vegetation (Piao et al., 2003), our results support the hypothesis that selection favors parrotbill in the warm southern range have larger body size to have the advantage in dense vegetation. Alternatively, competition between species in the same assemblage may affect evolution of body size (Olson et al., 2009). For instance, the distribution of *S. webbiana* overlaps with the sister species, ashy‐throated parrotbill (*S. alphonsiana*), in Southern‐west China (Shaner et al., 2015). The southern *S. webbiana* population have larger head size, which is positive correlated with body size, than sympatric *S. alphonsiana* and its northern population (Rao et al., 2018), which lives in colder places. Differences in body mass among closely related species help to reduce the chance of interspecific competition (Hespenheide, 1973) which could be an explanation of larger body size of *S. webbiana* found in warmer environments. Sexual selection may also determine body size, where larger males may enjoy an advantage in intrasexual competition involving advertisement display for mates and/or territory acquisition and defense (Price, 1984; Searcy, 1979). Body size could also be associated with frequency and duration of bird songs (Handford & Lougheed, 1991). Furthermore, the transmission of high‐frequency sound could be interfered by the dense vegetation (Morton, 1975). Because the lower latitudinal region of East Asia has the dense vegetation (Piao et al., 2003), the lower frequency call generated by larger body size might be able to transverse more efficiently in such environment. With accumulating evidence for the lack of relationship between body size/mass and temperature (Riemer et al., 2018), we suggest that thermoregulation might not play a significant role shaping body size variation in *S. webbiana*.

Similarly, no significant association was found between the tarsus length of *S. webbiana* and temperature may be largely attributed to forces other than climate, despite the fact that the naked tarsus is considered a major area of heat dissipation in cold environments (e.g., Midtgård, 1980). The tarsus (or tarsometatarsus) is a bone in the lower leg of birds that is homologous to the ankle (tarsus) and foot (metatarsal) bones of mammals, the length of which is suggested to be associated with force production, speed of movement, and energy consumption during locomotion (reviewed in Zeffer & Norberg, 2003). Tarsus length is highly associated with the body size of passerines (Senar & Pascual, 1997), presumably allowing them to maintain balance on the slender and unstable branches on which they perch (Grant, 1966; Schulenberg, 1983) and in turn affecting how they move through the environment to acquire food resources (Miles & Ricklefs, 1984). As *S. webbiana* is a small‐sized passerine (about 7–12 g) that forages in dense scrubs and thickets by picking seeds (Robson, 2020), the need to move on substrates such as twigs, leaves, and grass stems might impose constraints on tarsus length.

## 5 CONCLUSION

Ecogeographic rules, such as Allen's rule, Bergmann's rule, and Gloger's rule, predict how organism's morphology was shaped by the environments. These rules are mainly tested with species distributed in European and North America. However, due to the complex interplay of many evolutionary forces shaping morphological traits, a broad application of these ecogeographic rules across evolutionary lineages and biogeographic regions could lead to inaccurate forecasts. We suggest that before any of these rules is applied to predict phenotypic responses to climate change or the extreme climate event, their validity should be first established following the protocols used in this study (i.e., examining whole‐range climate–trait associations for the target taxa).

## AUTHOR CONTRIBUTIONS

**Chun‐Cheng Lee:** Formal analysis (equal); Visualization (supporting); Writing‐original draft (equal); Writing‐review and editing (equal). **Yuchen Fu:** Formal analysis (supporting); Visualization (lead); Writing‐original draft (supporting); Writing‐review and editing (supporting). **Chia‐fen Yeh:** Formal analysis (supporting); Resources (equal); Writing‐original draft (supporting). **Carol K. L. Yeung:** Writing‐original draft (supporting). **Hsin‐yi Hung:** Resources (equal). **Chiou‐Ju Yao:** Resources (equal). **Pei‐Jen Lee Shaner:** Formal analysis (equal); Writing‐original draft (equal); Writing‐review and editing (equal). **Shou‐Hsien Li:** Conceptualization (lead); Writing‐original draft (equal); Writing‐review and editing (equal).

## Supporting information

Supplementary Material

Table S1

## Data Availability

Measurements of specimens and climate data Dryad doi: https://doi.org/10.5061/dryad.573n5tb78.
