# Supplementary material for: Morphological variations in a widely distributed Eastern Asian passerine cannot be consistently explained by ecogeographic rules
Source: Ecol Evol. 2021 Oct 12;11(21):15249–60. doi: 10.1002/ece3.8208 (PMC8571641; doi:10.1002/ece3.8208)
Supplement: Supplementary file 1 — Supplementary Material [file ECE3-11-15249-s002.pdf]

Table S2. Correlations between 6 bioclimatic variables in plumage coloration data (n =41). Most of them were highly correlated with each other. Pearson correlation coefficient greater than 0.5 are in bold.

	bio1	bio5	bio6	bio12	bio16	bio17
bio1		**0.778**	**0.963**	**0.655**	0.388	**0.682**
bio5	**0.778**		**0.594**	0.451	0.199	**0.678**
bio6	**0.963**	**0.594**		**0.697**	0.435	**0.644**
bio12	**0.655**	0.451	**0.697**		**0.832**	**0.706**
bio16	0.388	0.199	0.435	**0.832**		0.229
bio17	**0.682**	**0.678**	**0.644**	**0.706**	0.229	

bio1 annual mean temperature (°C), *bio5* maximum temperature of warmest month (°C), *bio6* minimum temperature of coldest month (°C), *bio12* annual precipitation (mm), *bio16* precipitation of wettest quarter (mm), *bio17* precipitation of driest quarter (mm)

Table S3. Ranges of bioclimatic variables at Worldclim grids with vinous-throated parrotbill occurrence records (n=7,838).

Bioclimatic variable	Minimum	Maximum
bio1	-0.86°C	24.35°C
bio5	0.50°C	34.50°C
bio6	-27.10°C	12.60°C
bio12	307.83mm	2381.33mm
bio16	201.39mm	1262.26mm
bio17	6.00mm	246.45mm

bio1 annual mean temperature (°C), *bio5* maximum temperature of warmest month (°C), *bio6* minimum temperature of coldest month (°C), *bio12* annual precipitation (mm), *bio16* precipitation of wettest quarter (mm), *bio17* precipitation of driest quarter (mm)

Table S4. Ranges of bioclimatic variables of localities where skin specimens for morphometric (n=114) and plumage coloration (n=41, in brackets) analysis were collected.

Bioclimatic variables	Minimum	Maximum
bio1	2.98°C (2.98°C)	20.65°C (17.71°C)
bio5	20.95°C (15.7°C)	33.8°C (33.23°C)
bio6	-23.64°C (-23.64°C)	8.2°C (4.4°C)
bio12	471.97mm (471.97 mm)	1837.2 mm (1634.76 mm)
bio16	297.63 mm (326.88 mm)	943.39 mm (990.58 mm)
bio17	10.06 mm (10.06 mm)	197.78 mm (189.2 mm)

bio1 annual mean temperature (°C), *bio5* maximum temperature of warmest month (°C), *bio6* minimum temperature of coldest month (°C), *bio12* annual precipitation (mm), *bio16* precipitation of wettest quarter (mm), *bio17* precipitation of driest quarter (mm)

Table S5. Summary statistic of morphometric traits and plumage coloration. Morphometric traits (culmen length, beak volume, tarsus length and wing length) were summarized by specimen of 155 males and 135 females. Plumage coloration (hue, chroma and brightness) was summarized by specimen of 43 males and 58 females.

Male	Culmen length (mm)	Beak volume (mm^3)	Tarsus length (mm)	Wing length (mm)	Hue	Chroma	Brightness
maximum	9.12	18.12	21.92	50.99	0.0695	0.551	11
minimum	7.51	9.64	19.17	39.59	0.0359	0.475	6.09
mean	8.39	13.4	20.2	45.9	0.0534	0.509	8.44
stander deviation	0.375	1.7	0.526	2.675	0.00883	0.0253	1.39
Female	Culmen length (mm)	Beak volume (mm^3)	Tarsus length(mm)	Wing length (mm)	Hue	Chroma	Brightness
maximum	9.03	16.15	21.16	49.35	0.0648	0.547	11.4
minimum	7.59	9.64	19.08	38.9	0.0355	0.463	6.23
mean	8.17	12.7	20	44.4	0.0546	0.499	8.94
stander deviation	0.33	1.402	0.497	2.473	0.00855	0.0227	1.47

Table S6. Results of ANCOVAs examining relationships between residual of morphometric traits and climatic variables. Residual of morphometric traits served as body size independent traits. Sex was treated as a covariate. Lower limit (2.5%) and upper limit (97.5%) show the 95% confidence interval (CI) of slope from 10,000 replicates. Statistical significance was determined by whether the 95% CI of the estimated slope from resampled data sets includes zero. Significant results are in bold.

		Confidence Interval of Slope		
response	fixed effect	lower limit	median	upper limit
Residual of culmen length	**bio1**	**-0.0400**	**-0.0239**	**-0.0079**
	bio5	-0.0262	-0.0111	0.0039
	**bio6**	**-0.0253**	**-0.0162**	**-0.0071**
Residual of beak volume	**bio1**	**-0.1147**	**-0.0665**	**-0.0183**
	**bio5**	**0.0128**	**0.0614**	**0.1101**
	**bio6**	**-0.0909**	**-0.0628**	**-0.0347**
Residual of tarsus length	bio1	-0.0189	-0.0029	0.0129
	bio5	-0.0223	-0.0034	0.0156
	bio6	-0.0115	-0.0022	0.0071

bio1 annual mean temperature (°C), *bio5* maximum temperature of warmest month (°C), *bio6* minimum temperature of coldest month (°C)

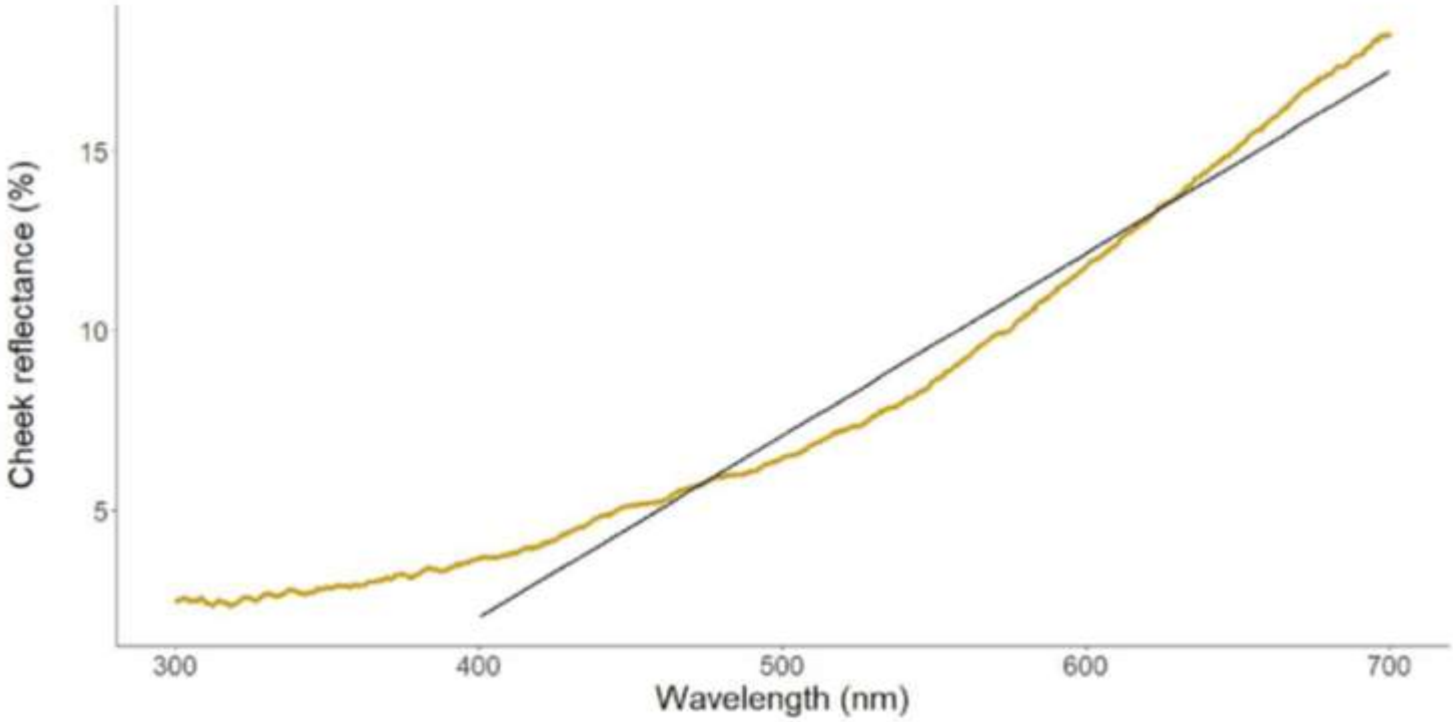

Figure S1. The wavelength reflectance of the cheek plumage. The dark yellow line represents the reflectance percentage at a certain wavelength; the black line is the regression line at 400 to 700 nm, the slope of which was used as the hue of the plumage.

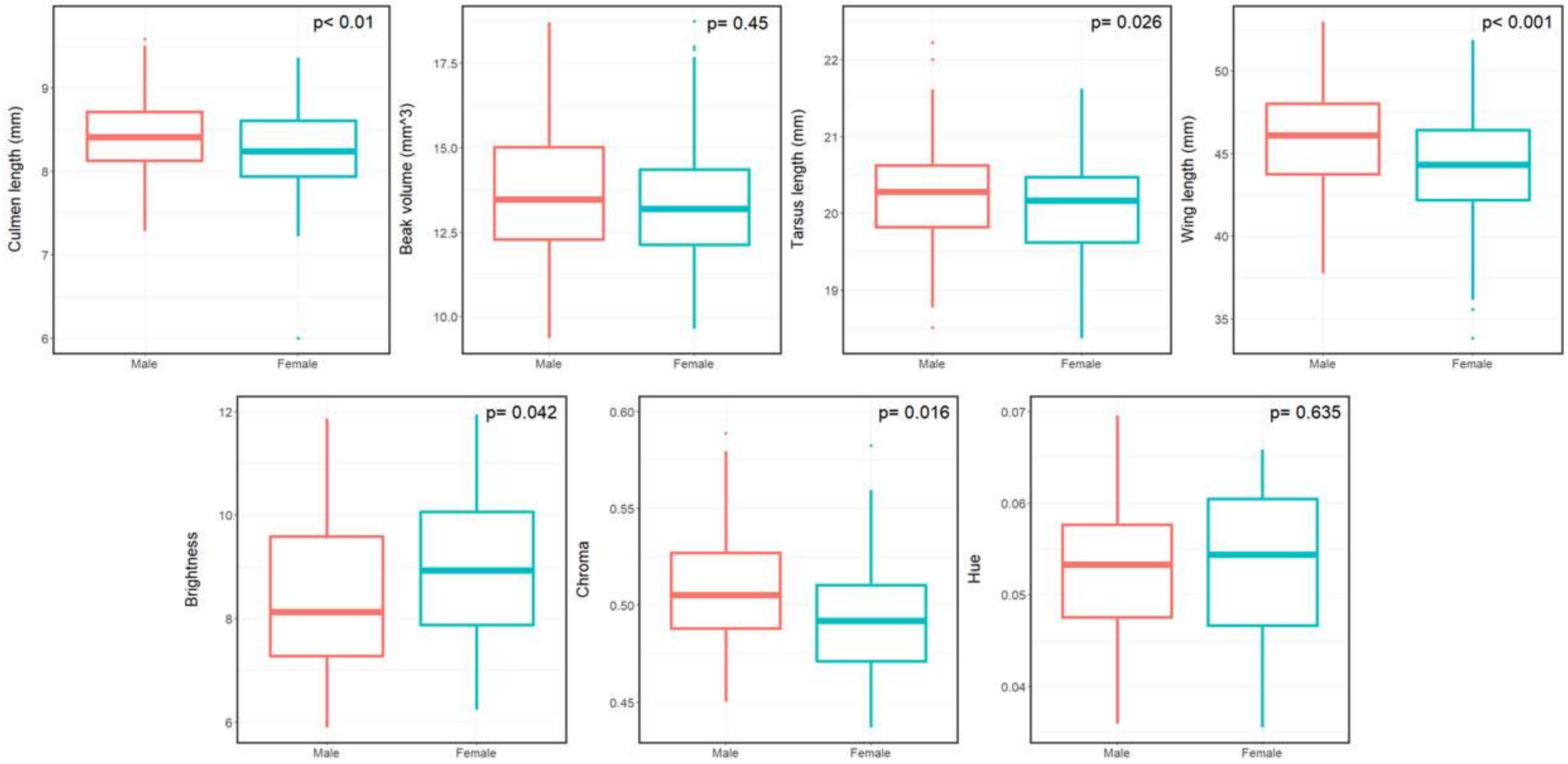

Figure S2. Boxplots of morphometric traits (n= 290) and plumage coloration (n= 101) in specimen collections. The p value showed the significance of difference between male and female.
